# Decomposition and Changes in In Vivo Post‐HA Filler Injection: A Review

**DOI:** 10.1111/jocd.16652

**Published:** 2024-10-28

**Authors:** Gi‐Woong Hong, Jovian Wan, Kathleen Chang, Youngjin Park, Kyu‐Ho Yi

**Affiliations:** ^1^ Samskin Plastic Surgery Clinic Seoul Korea; ^2^ Asia Pacific Aesthetic Academy Hong Kong; ^3^ Harmony Aesthetic Clinic Adelaide Australia; ^4^ Obliv Clinic Incheon Korea; ^5^ Division in Anatomy and Developmental Biology, Department of Oral Biology, Human Identification Research Institute, BK21 FOUR Project Yonsei University College of Dentistry Seoul Korea; ^6^ Maylin Clinic (Apgujeong) Seoul Korea

**Keywords:** biocompatible materials, dermal fillers, drug‐related side effects and adverse reactions, hyaluronic acid, tissue engineering

## Abstract

**Background:**

Hyaluronic acid (HA) fillers are widely used in aesthetic medicine, but their in vivo behavior and long‐term effects are not fully understood.

**Aims:**

To review the decomposition and changes occurring in the body following HA filler injections, focusing on crosslinking agents, degradation processes, and tissue responses.

**Methods:**

This review analyzed oxidative and enzymatic degradation processes of HA fillers, evaluated the impact of 1,4‐Butanediol Diglycidyl Ether (BDDE) crosslinking, and examined histological changes post‐injection.

**Results:**

Uncrosslinked HA degrades rapidly due to endogenous hyaluronidase, while crosslinked HA undergoes slower degradation via free radicals and hyaluronidase. Complete cross‐linking (C‐MoD) showed better durability compared to partially cross‐linked BDDE (P‐MoD). The concept of modification efficiency (MoE) was proposed to optimize filler safety and viscoelastic properties. Histological analysis revealed collagen capsule formation and autologous tissue replacement, affecting long‐term outcomes. The degree of chemical modification (MoD) influences filler durability and safety, with concerns raised about potential delayed immune reactions from accumulated pendent BDDE.

**Conclusions:**

Clinicians should consider injection site, tissue conditions, and filler properties for safe and effective HA filler use. Emphasizing thorough BDDE removal and optimal crosslinking can enhance treatment safety and efficacy. The balance between achieving desired viscoelastic properties and minimizing potential risks is crucial. Future studies should include diverse ethnic groups to validate findings and further explore long‐term tissue responses to HA fillers.

## 1 Introduction

Uncrosslinked natural hyaluronic acid (HA) in its natural state is quickly degraded and eliminated by the body's endogenous enzyme, hyaluronidase, within a few days [1, 2]. Therefore, to extend the duration of HA fillers, a crosslinking agent must be used to create crosslinks between HA molecules, regardless of whether the filler emphasizes natural entanglement with minimal crosslinking agent as in biphasic HA fillers, or uses increased amounts of chemical crosslinking agents as in monophasic HA fillers. The most commonly used crosslinking agent is BDDE (1,4‐Butanediol Diglycidyl Ether). BDDE has epoxide groups at both ends, which bind to the hydroxyl groups (‐OH) of HA molecules, forming ether bonds [3].

**FIGURE 1:**
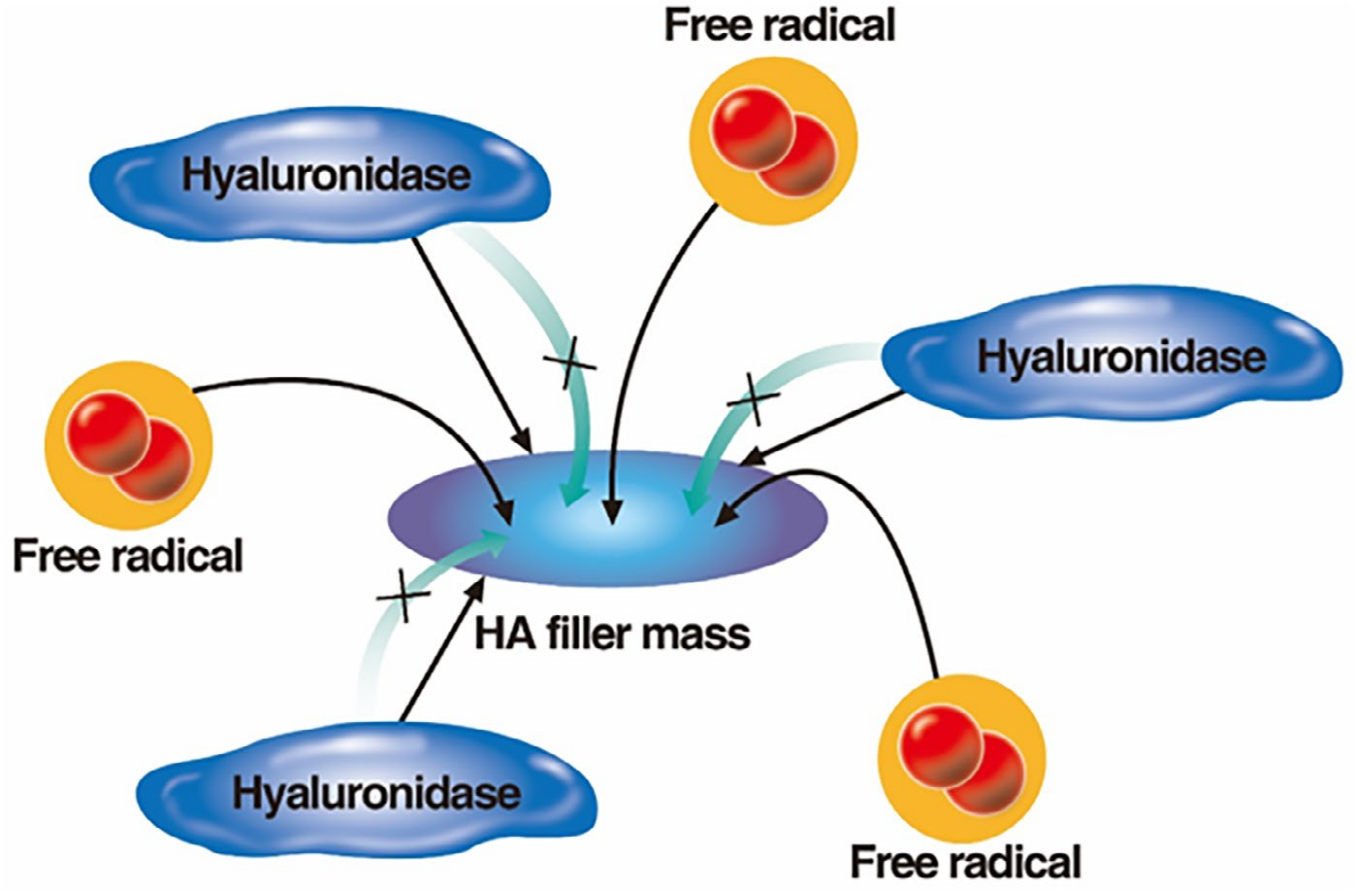
Difference of action sites between free radical & hyaluronidase.

Once injected into the body, HA fillers crosslinked with BDDE undergo two main degradation processes. The first is oxidative degradation caused by free radicals generated during mechanical injury to tissues by needles or cannulas during filler injection. The insertion and manipulation of needles or cannulas induce acute tissue inflammation, temporarily increasing free radical activity at the injection site. Free radicals, being small, can freely move into the pores between filler particles and penetrate the filler, breaking down its structure (Figure 1) [1, 4].

The second process is enzymatic degradation caused by hyaluronidase, an enzyme that degrades HA. However, because hyaluronidase has a large molecular size, it can only act on the surface of the HA filler mass (Figure 1) [4, 5, 6, 7].

Free radicals and hyaluronidase degrade long, large HA chains (polysaccharides) into smaller HA units (oligosaccharides). These smaller units undergo metabolic breakdown within cells or lymph nodes, eventually entering the circulatory system and being filtered out by the liver and kidneys [3, 7].

As HA fillers undergo these two degradation processes and are metabolized, there is generally no need to be concerned about their elimination. However, BDDE used as a crosslinking agent in the filler product cannot be degraded by enzymes and must be eliminated through other processes such as hydrolytic degradation. BDDE molecules that are not bound to HA molecules and exist independently can become toxic artifacts that trigger immune reactions. Additionally, the process by which BDDE bound to HA molecules is handled in the body varies depending on the type of binding [3, 8].

First, we will examine the different binding types of BDDE to HA in the final HA filler product and discuss the clinical significance and in vivo handling processes of each type.

## 2 Degree and Efficiency of Chemical Modification

Type of chemical modification (complete or incomplete cross‐linked BDDE).

**FIGURE 2:**
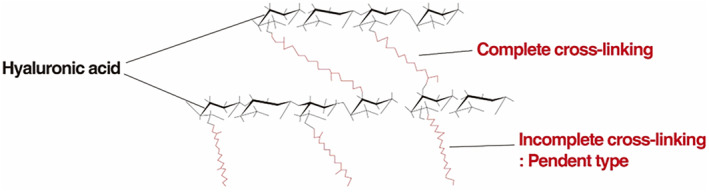
Type of chemical cross‐linking.

BDDE, used to cross‐link HA molecules, can be categorized into those that react with the HA backbone and those that do not. Among the reacted BDDE, if both ends of the ether groups are linked to HA, it is termed “fully reacted BDDE,” indicating complete cross‐linking. Conversely, if only one ether group is connected to HA, it is considered “partially cross‐linked” and is known as the “pendant type,” as it hangs from the hyaluronic acid chain like a pendant (Figure 2) ([2, 3, 8]).

BDDE that does not react with HA and undergoes hydrolysis with water is termed “deactivated cross‐linker,” while BDDE that does not react with water remains as “residual cross‐linker.” Hydrolyzed BDDE breaks down into glycerol and butanediol and is excreted, with some being eliminated through urine. Since hydrolyzed BDDE is nontoxic and non‐genotoxic, ensuring that residual BDDE is sufficiently removed during the HA filler manufacturing process minimizes toxicity concerns [3, 7, 8, 9].

If residual BDDE is not adequately removed before injection, its epoxide groups can chemically react with body tissues. Although residual BDDE has not shown carcinogenic effects in animal studies, it has demonstrated mutagenic potential. Therefore, residual BDDE must be thoroughly removed through washing and dialysis during the filler manufacturing process to undetectable levels. Regulatory standards typically limit detectable residual BDDE in HA fillers to a maximum of 2 ppm (0.002 mg of BDDE per 1 mL of HA gel) [3, 7, 8, 9, 10].

BDDE cross‐linked with HA molecules is considered safe and does not chemically react when injected into the body. It is also known that BDDE left over from the slow degradation of HA fillers is safely eliminated without reactivation.

However, concerns have arisen regarding the long‐term safety of pendent‐type BDDE, which is not fully cross‐linked. Previous studies reported that these partially cross‐linked BDDEs lose their toxicity upon reacting with water post‐injection. Yet, with the increasing use of HA fillers with high cross‐linking to enhance viscoelasticity and the frequent administration of large amounts, the accumulation of pendent‐type BDDE in the body has increased. This accumulation, which remains after HA filler degradation, is suspected to be associated with delayed immune reactions and related side effects (Delayed immunologic complications due to injectable fillers by unlicensed practitioners: Our experience and a review of the literature, Delayed‐onset nodules secondary to smooth cohesive 20 mg/mL hyaluronic acid filler: Cause and management, Adverse reactions to injectable soft tissue permanent fillers).

Therefore, HA fillers with lower degrees of chemical cross‐linking are considered relatively safer when aiming for similar levels of viscoelasticity. In the past, before this knowledge was widespread, HA fillers with high cross‐linking were released, causing numerous issues and eventually being withdrawn from the market. (Inflammatory nodules following soft tissue filler use: A review of causative agents, pathology and treatment options).

### 2.1 Degree of Chemical Modification (MoD)

Whether the HA molecules are completely bonded with BDDE, known as C‐MOD (Complete cross‐link modified disaccharides), or partially bonded, known as P‐MOD (Pendent modified disaccharides), they are collectively referred to as T‐MOD (total modified disaccharides) since they all involve modification through BDDE binding [11].

**FIGURE 3:**
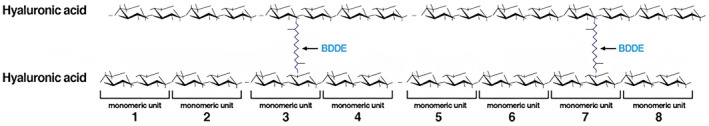
Cross‐linking ratio of HA filler based on chemical cross‐linking.

The proportion of modified HA molecules within a filler, indicating the degree of cross‐linking with BDDE, is referred to as the cross‐linking ratio or the Degree of Chemical Modification (MoD). This ratio represents how many of the 100 HA monomeric units are bonded with BDDE (Figure 3).

Generally, a higher T‐MOD indicates a firmer filler with higher viscoelastic properties. More precisely, even fillers with the same T‐MOD can exhibit different viscoelasticity due to varying proportions of C‐MOD within the T‐MOD. Fillers with a higher proportion of C‐MOD, signifying complete cross‐linking, tend to be firmer, more resistant to enzymatic degradation, and longer‐lasting [3, 4].

The MoD value varies among HA filler products. Products with similar viscoelastic properties but lower MoD values use fewer cross‐linking agents to achieve the same effect. Biphasic HA fillers typically exhibit lower MoD values because they rely on minimal BDDE and natural entanglement for firmness. However, among monophasic HA fillers with similar viscoelastic properties, differences in MoD values often stem from variations in P‐MOD, reflecting BDDE bonded to only one side of the HA molecule [3, 4].

The overall MOD value, including P‐MOD, is measured using Nuclear Magnetic Resonance (NMR), a complex and challenging process. Therefore, most filler manufacturers focus on the structure formed by completely cross‐linked BDDE and calculate only the C‐MOD value using SEC/MS methods, as they believe pendent BDDE has minimal impact on the filler structure.

**FIGURE 4:**
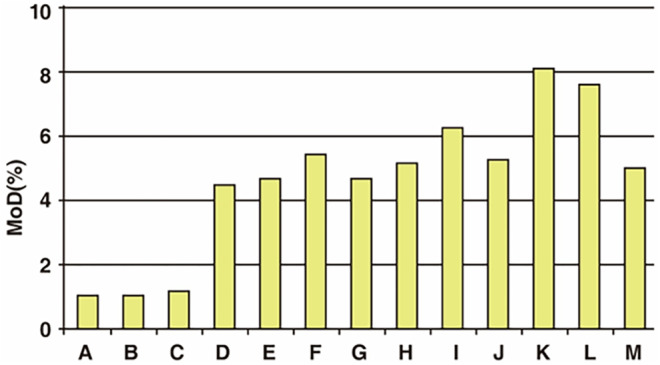
Determination of modification degree of HA fillers based on chemical cross‐linking.

Consequently, the MoD values reported by manufacturers are based on the amount of completely cross‐linked BDDE used in production. Typically, the standard MoD values presented by companies, based on complete cross‐linking, range between 1% and 10% (Figure 4) [2, 3].

### 2.2 Efficiency of Chemical Modification (MoE)

Another important consideration is that the ratio of completely cross‐linked (C‐MOD) to partially cross‐linked (P‐MOD) HA molecules can impact the physical properties and safety of the filler. Even products with similar viscoelasticity may have different MoD values depending on the manufacturing process. It is generally recognized that using less BDDE, the cross‐linking agent, is safer. Recently, concerns have been raised about the potential harmfulness of P‐MOD. Therefore, it is now argued that, in addition to the MoD value, we should consider the modification efficiency (MoE), which indicates how much BDDE was used to achieve a certain level of viscoelasticity. MoE can be calculated as MoE = gel strength/MoD [3, 4].

Ideally, we would like to know the total MOD value, including the ratios of C‐MOD and P‐MOD for all HA filler products. However, as previously mentioned, measuring these values is difficult, and typically, only C‐MOD values are provided by manufacturers, making it hard to determine the extent of P‐MOD. Therefore, the ratio of complete to pendent cross‐linking in each HA filler product can only be inferred from clinical outcomes [3, 4].

As discussed earlier, BDDE bound to HA molecules remains after the HA is degraded by enzymes and must be excreted through the body's metabolic processes. Since BDDE is inherently toxic, problems can arise if the amount of BDDE accumulated exceeds the body's capacity to eliminate it. This concern has been highlighted in several studies. Among monophasic HA fillers, products that use relatively high amounts of BDDE and exhibit similar viscoelastic properties with little difference in C‐MOD values sometimes show a higher incidence of delayed onset nodules months after injection. Persistent clinical symptoms of delayed tissue reactions, despite treatment, can suggest a higher P‐MOD value due to pendent BDDE [10].

The amount of BDDE used in compliance with safety standards determines the overall BDDE content based on the amount of filler used over a certain period. The FDA, considering the potential for immune reactions from accumulated cross‐linkers, recommends a maximum of 20 mL of HA filler per year for a 60 kg adult. Staying within this limit reduces the likelihood of issues, but HA fillers with less BDDE, even with similar viscoelasticity, are safer regarding BDDE‐induced immunological toxicity, making MoE a useful measure [12, 13].

Korean domestic HA filler manufacturers provide MoD values based on C‐MOD, excluding P‐MOD, so MoE is calculated using the gel strength and C‐MOD values of each product. Typically, products with higher MoE have lower MoD values compared to other products with similar viscoelasticity, indicating a technically superior product made with less cross‐linker.

**FIGURE 5:**
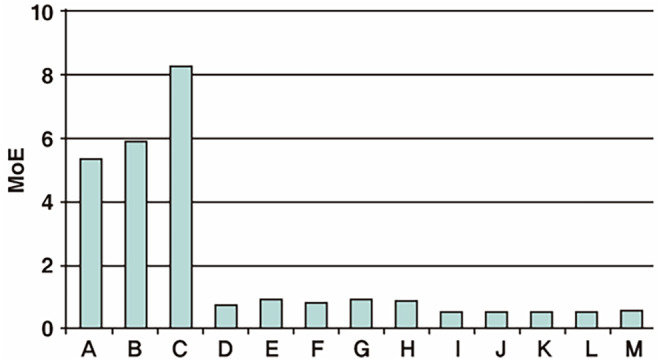
Determination of modification efficiency of HA fillers based on chemical cross‐linking.

Currently, the MoE values of products on the market suggest that biphasic HA fillers generally perform better than monophasic fillers, due to the characteristics of their manufacturing process (Figure 5) [14, 15].

### 2.3 Histological Changes in Human Tissue Response to HA Fillers

After the injection of fillers into the human body, the biological tissue reacts to the foreign substance with responses such as inflammation, swelling, capsule formation, biostimulatory effects, and foreign body reactions [14, 16].

Initially, when an unfamiliar foreign substance enters the body, plasma proteins adhere to the surface of the material, triggering a tissue response. This process activates phagocytes such as monocytes and macrophages, which then surround the foreign substance. Subsequently, these phagocytes fuse to form multinucleated giant cells. As collagen is generated, a dense collagen capsule that fully encases the foreign material is formed, known as a foreign body reaction [14, 16].

Because HA is a natural polymer, it typically induces fewer foreign body reactions compared to synthetic polymers like PLLA or PCL, which are known to cause more pronounced foreign body reactions and greater collagen production when used in longer‐lasting fillers [17, 18, 19].

Microscopic examination of tissue reactions to injected fillers reveals that a capsule forms around the injected filler, usually observable from 4 weeks post‐injection. The thin layer seen immediately after the procedure is thought to be a structure formed by compressed subcutaneous tissue.

After 4 weeks, a lattice structure is created by fibroblasts, new blood vessels, and collagen regeneration. As the HA filler gradually degrades and is absorbed, this structure is progressively replaced by autologous tissue composed of fibroblasts, connective tissue, adipocytes, and blood vessels [17, 18, 19].

Examining changes in Type III collagen, which is observed in the early stages of wound healing, at the injection site of HA fillers shows a rapid increase during the first 8 weeks. After 4 weeks, this collagen accounts for about 13.8% of the filler volume, reaching its peak at approximately 32 weeks, where it constitutes about 21.5% of the total filler volume [8, 14].

These observations confirm that HA fillers injected into the body are partially replaced by autologous tissue due to the tissue reaction. Although the duration of HA fillers can vary depending on the area and layer of the face, it is now believed that HA fillers can last for more than one to two years. Patients are informed that even after a longer period, the portion replaced by autologous tissue does not fully revert to its original state before the filler was injected [18, 20].

In some cases, the shape created by the HA filler persists longer than expected and does not diminish significantly, suggesting a strong tissue reaction. This response leads to effective replacement of the filler mass by autologous tissue and the formation of a thick capsule around the filler mass due to collagen regeneration, which prevents the degradation and absorption of the HA filler by hyaluronidase acting only on the surface of the mass [21].

While such replacement and thick capsule formation may seem beneficial due to prolonged filler retention, excessively thick capsules can cause biofilm infections and are not necessarily advantageous. An optimal tissue response that induces moderate capsule formation is considered safer and more effective [13, 16, 22, 23].

A proper tissue response post‐filler injection not only helps maintain the filler volume but also extends its duration. Unlike in the past, when it was believed that HA fillers would completely disappear after a few months, it is now understood that as the body's tissue cells change over time, the autologous tissue generated by the filler also changes. Therefore, fillers can be replenished as needed to maintain the desired appearance [2, 12, 24].

Currently, patients report high satisfaction with the long‐lasting effects of filler injections, even without frequent touch‐ups, and they do not completely revert to their pre‐injection appearance over time.

Therefore, when selecting and injecting HA filler products, practitioners must consider factors such as the injection site, the patient's skin and soft tissue condition, the injection layer, the physical properties of the filler, the injection technique, post‐injection external stimuli, and the degree of tissue reaction within the body. These factors can influence the volume change and duration of the filler [13, 19].

## 3 Relationship Between Shape Changes and Physical Properties of HA Fillers After Injection

HA fillers, being viscoelastic substances, can be injected through thin tubes like needles or cannulas. After injection, due to their viscoelastic properties, they restore their original shape and function as fillers. The primary purposes of fillers can be divided into two categories: first, to correct depressions in the dermis for wrinkle correction, and second, to increase the volume of facial skin and soft tissues for volumizing. Clinically, there are now more types of fillers aimed at increasing facial volume [25, 26, 27, 28].

Analyzing how the shape of a filler changes after being injected to enhance volume reveals that the hydrophilic nature of HA fillers causes an increase in volume during the initial 4–8 weeks. Typically, the volume created by the injected filler peaks at approximately 1.8 times the initial volume after 4 weeks. This volume gradually decreases, reaching about 75% of the initial volume by 16 weeks and remaining relatively stable between 16 and 64 weeks. The maximum height of the shape created by the HA filler reduces to about two‐thirds of its initial height due to tissue pressure during the first 4 weeks, and then maintains a relatively stable height. The width of the shape increases similarly to the overall volume, reaching about 1.8 times the initial width during the first 4 weeks. It remains wider than immediately after the procedure for the next 4–32 weeks before gradually decreasing to a similar width as the initial shape after 64 weeks [14].

**FIGURE 6:**
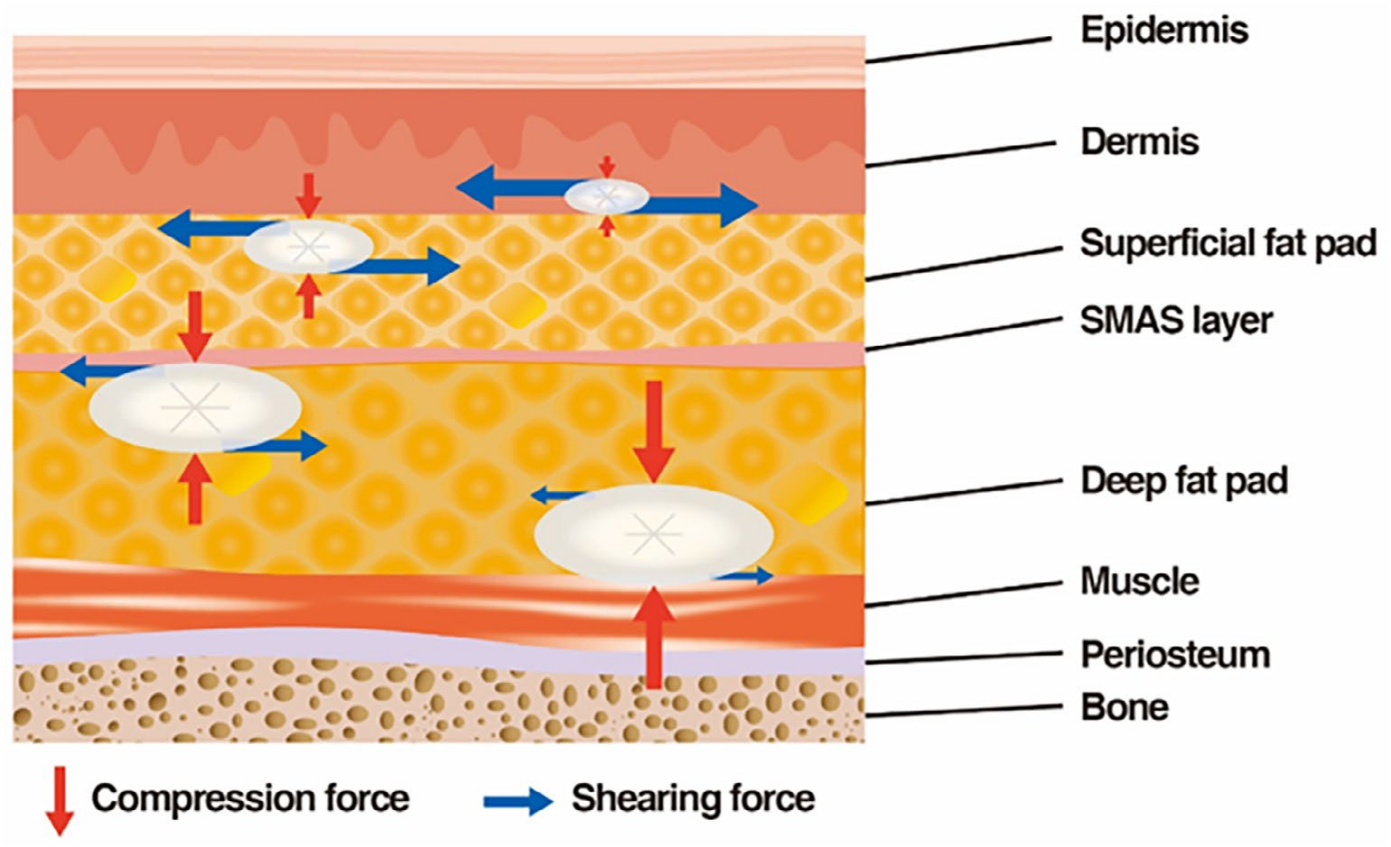
Difference of dynamic forces that contribute to deformation of HA fillers between superficial and deep plane.

When HA fillers are used to increase facial volume, they are often injected into layers deeper than the SMAS. These deeper layers experience less horizontal movement due to facial muscles but are subjected to significant external pressure from surrounding ligaments and the SMAS. Clinically, softer fillers with low elasticity can easily create the desired shape in this space but may not maintain it well under pressure. In contrast, firmer fillers with high viscoelasticity require more force to inject and shape but maintain their form better once in place (Figure 6). While firmer fillers are preferable for maintaining shape in deeper layers, they can also increase the risk of complications such as a foreign body sensation, impaired blood circulation, and tissue reactions due to the increased pressure on surrounding tissues. Therefore, it is important to choose a filler with appropriate elasticity for the injection layer [14, 29, 30].

For wrinkle correction or smoothing the skin, fillers are injected into layers closer to the skin, where they encounter the fibrous parts of facial muscles. These areas experience more horizontal stretching and contraction due to facial expressions, rather than vertical pressure. Hence, flexible fillers that can stretch and return to their original shape without discomfort during facial movements are suitable for these layers (Figure 5). Soft, low‐viscoelasticity fillers are needed for even distribution, especially around the eyes, where fillers need to spread smoothly under or just below the skin for a smooth surface. However, excessively soft fillers may deform easily and not return to their original shape, making them less desirable [29, 30].

The appropriate physical properties of fillers should be chosen based on the injection site, the condition of the patient's face, and the purpose of the procedure. Other considerations include anticipating the need for active molding. For example, in nasal filler procedures for asymmetric noses, molding should be performed concurrently with filler injection to achieve a symmetrical shape. It is important to predict the exposure to external pressure or facial movements. For example, the bridge of the nose can be affected by facial expressions or pressure from glasses, which should be discussed with the patient beforehand. Additionally, consider the pressure of skin and soft tissues on the injected area. For instance, in cases of severe nasal depression, the injected filler might spread due to tissue pressure. Pinching the skin to assess adherence can help in choosing a firmer filler or planning for additional corrections [1, 21, 30].

Knowing the volume change characteristics of each HA filler product is essential. Products pre‐hydrated during manufacturing are less likely to swell post‐injection. Biphasic fillers, due to their structure, absorb less water and swell less than monophasic fillers. However, even among monophasic fillers, those with higher cross‐linking absorb less water and swell less. HA fillers containing free HA may show initial volume increase due to water absorption, but this diminishes as free HA is absorbed, leaving only cross‐linked HA. This can cause dissatisfaction as the initial volume decreases within a few weeks [7].

Conversely, fillers with a high amount of pendent type BDDE might swell excessively and for a prolonged period. This unexplained swelling compared to other products could be due to incomplete cross‐linking of BDDE, suggesting a need for clinical observation before increasing usage. By considering these factors, clinicians can select the most appropriate filler for each patient's needs, ensuring effective and long‐lasting results. (Global Aesthetics Consensus: Avoidance and Management of Complications from Hyaluronic Acid Fillers―Evidence‐ and Opinion‐Based Review and Consensus Recommendation).

## 4 Discussion

The review on the decomposition and changes in the body post‐HA filler injection highlights the critical role of crosslinking agents, particularly BDDE, in enhancing the longevity and effectiveness of HA fillers. The oxidative and enzymatic degradation processes are crucial in understanding the metabolic pathways and eventual elimination of HA fillers from the body. While BDDE‐crosslinked HA fillers are generally considered safe due to their controlled degradation and minimal toxicity of fully reacted BDDE, the presence of residual and pendent BDDE poses potential long‐term safety concerns. The review underscores the importance of thorough removal of residual BDDE during manufacturing to prevent mutagenic potential and adverse immune reactions [3, 14, 31].

Furthermore, the differentiation between fully and partially cross‐linked BDDE provides insights into the physical properties and safety profiles of HA fillers. Fillers with a higher proportion of C‐MOD exhibit better viscoelasticity and durability, making them preferable for longer‐lasting results. However, the accumulation of pendent type BDDE, due to its incomplete cross‐linking, raises concerns about delayed immune reactions and associated side effects. This highlights the need for a balanced approach in HA filler formulation, optimizing the degree of chemical modification (MoD) to achieve the desired viscoelastic properties while minimizing potential risks. The concept of modification efficiency (MoE) emerges as a valuable metric, encouraging the use of minimal BDDE for optimal gel strength, thereby enhancing the overall safety profile of HA fillers [22, 32].

The paper emphasizes the histological changes in tissue response to HA fillers, providing a comprehensive understanding of how these materials interact with biological tissues. The formation of collagen capsules and the gradual replacement of filler material with autologous tissue demonstrate the dynamic process of filler integration and degradation. The clinical implications of these histological changes are significant, as they influence long‐term outcomes and patient satisfaction. Understanding the tissue response and the factors affecting filler longevity allows clinicians to make informed decisions about filler selection and application, ensuring both efficacy and safety in cosmetic procedures [12, 33].

## Author Contributions

All authors have reviewed and approved the article for submission. Conceptualization – Gi‐Woong Hong, Kyu‐Ho Yi, Jovian Wan, Kathleen Chang, Youngjin Park. Writing – Original Draft Preparation, Gi‐Woong Hong, Jovian Wan, Kathleen Chang, Youngjin Park. Writing – Review and Editing, Gi‐Woong Hong, and Kyu‐Ho Yi. Visualization – Gi‐Woong Hong and Kyu‐Ho Yi. Supervision – Gi‐Woong Hong and Kyu‐Ho Yi.

## Disclosure

The authors have nothing to report.

## Conflicts of Interest

The authors declare no conflicts of interest.

## Data Availability

The data ais available on request to corresponding author.
